# Low-Medium and High-Intensity Inspiratory Muscle Training in Critically Ill Patients: A Systematic Review and Meta-Analysis

**DOI:** 10.3390/medicina60060869

**Published:** 2024-05-26

**Authors:** Irini Patsaki, Alexandros Kouvarakos, Ioannis Vasileiadis, Georgios A. Koumantakis, Eleni Ischaki, Eirini Grammatopoulou, Anastasia Kotanidou, Eleni E. Magira

**Affiliations:** 1 Laboratory of Advanced Physiotherapy, Physiotherapy Department, School of Health & Care Sciences, University of West Attica (UNIWA), 12243 Athens, Greecegkoumantakis@uniwa.gr (G.A.K.);; 2 1st Critical Care Department, General Hospital of Athens “Evagelismos”, National and Kapodistrian University of Athens, 11527 Athens, Greece

**Keywords:** inspiratory muscle training, weaning, ICU, maximum inspiratory pressure, mechanical ventilation, critically ill

## Abstract

*Background and objectives*: Mechanical ventilation is often used in intensive care units to assist patients’ breathing. This often leads to respiratory muscle weakness and diaphragmatic dysfunction, causing weaning difficulties. Inspiratory muscle training (IMT) has been found to be beneficial in increasing inspiratory muscle strength and facilitating weaning. Over the years, different protocols and devices have been used. *Materials and Methods*: The aim of this systematic review and meta-analysis was to investigate the effectiveness of low-medium (LM-IMT) and high-intensity (H-IMT) threshold inspiratory muscle training in critically ill patients. A systematic literature search was performed for randomized controlled trials (RCTs) in the electronic databases Google Scholar, PubMed, Scopus, and Science Direct. The search involved screening for studies examining the effectiveness of two different intensities of threshold IMT in critically ill patients published the last 10 years. The Physiotherapy Evidence Database (PEDro) scale was chosen as the tool to assess the quality of studies. A meta-analysis was performed where possible. *Results*: Fourteen studies were included in the systematic review, with five of them having high methodological quality. *Conclusions*: When examining LM-IMT and H-IMT though, neither was able to reach statistically significant improvement in their maximal inspiratory pressure (MIP), while LM-IMT reached it in terms of weaning duration. Additionally, no statistical difference was noticed in the duration of mechanical ventilation. The application of IMT is recommended to ICU patients in order to prevent diaphragmatic dysfunction and facilitate weaning from mechanical ventilation. Therefore, further research as well as additional RCTs regarding different protocols are needed to enhance its effectiveness.

## 1. Introduction

Respiratory support using invasive mechanical ventilation (MV) is the cornerstone of medical care in the intensive care unit (ICU). However, its prolonged application has been found to lead to serious complications such as ventilator-associated pneumonia, lung injury, and diaphragmatic dysfunction [1,2]. It has been found that exposure to controlled MV for 18–69 h produces significant diaphragmatic atrophy and changes in myofibrillar length [3]. Weakness of the breathing muscles due to their atrophy and structural dysfunction leads to an inability to release from MV [4]. One-third of patients that received MV for a period of 7 days or more have presented weakness and a decrease in inspiratory muscle endurance shortly after successful weaning [5]. In addition, increased dyspnea has been observed both during rest and during exercise, which has an inhibitory effect on the functional recovery of these patients [3,6]. A longer stay under MV increases the risk of complications, such as infections and neuromuscular syndromes, and also increases the mortality rate [6,7].

Inspiratory muscle training (IMT) is an emerging form of therapy with promising results for reducing diaphragmatic weakness in ICU patients. It consists of a wide range of techniques (through removable devices like flow resistance or threshold or through the ventilator’s triggering settings). However, the most common approach is through threshold loading [8]. This device has a spring-loaded one-way valve that provides titratable inspiratory resistance during the inspiratory effort of the participant [8]. More recently, we have seen the implementation of electronic devices that apply a tapered flow resistive load, which seems to allow larger volume expansion and higher inspiratory flow rates [9].

Recent systematic reviews have revealed that this intervention is feasible, well tolerated by the patient, improves respiratory muscle strength and respiratory function, accelerates weaning, and contributes to a possible reduction in ICU length of stay and shorter use of noninvasive respiratory support [9,10].

However, the above reviews included heterogeneous studies regarding time of intervention initiation, the duration of IMT application, and the technique used. Additionally, the last systematic review published in 2018 [9] highlighted the need to further investigate the beneficial effects of the specific programs in clinical indicators. It is in this light that the present review was conducted, in which an effort will be made to investigate any effect that could have different intensities of IMT in critically ill patients.

## 2. Materials and Methods

The purpose of this systematic review was to present the effects of different training intensities of inspiratory muscle training in ICU patients.

A systematic review and meta-analysis were conducted according to the Preferred Reporting Items for Systematic Reviews and Meta-Analyses (PRISMA) 2020 guidelines [11], and the methodological quality assessment of the clinical trials was conducted according to the PEDro scale [12].

### 2.1. Eligibility

The criteria for inclusion of studies in this systematic review and meta-analysis were as follows: (1) RCT study design, (2) participants 18 years of age or older, (3) critically ill patients under mechanical ventilation for >48 h, (4) threshold or tapered flow resistive inspiratory muscle training intervention, and (5) written in English and published during the last 10 years.

The exclusion criteria from the research study were (1) the inspiratory muscle training being performed via ventilation, (2) protocols, systematic reviews, publications of session lectures, study protocols, posters, cohort studies, case studies, and abstracts, as they cannot be studied systematically, (3) the characteristics of the inspiratory training program not being described well in detail, and (4) papers which were not fully extracted.

### 2.2. Search Strategy

To identify eligible studies, a comprehensive search was performed from January 2023 to July 2023 in the following online databases: Google Scholar, PubMed, Scopus, and Science direct. During the search, the following keywords were used regarding the intervention applied: “Inspiratory muscle training” OR “Respiratory muscle training”. These were used in combination with terms regarding population (“Intensive Care Unit” OR “Critically ill” OR “mechanically ventilated”) and with terms regarding outcomes (“Maximal Respiratory Pressure” AND “Weaning” AND “Mechanical Ventilation”). These were used to create the different search strategies.

### 2.3. Study Selection and Extraction

A thorough review of the titles and abstracts of studies published in the databases used was performed. For those studies that met the criteria according to title and abstract, a full analysis was performed for further content review. Additionally, the reference lists of the pertinent literature were searched for potentially relevant articles in English. The search strategy was carried out by two authors (I.P. and A.K.) independently, and any differences were resolved by consensus between the two reviewers or by a third when needed.

A predesigned data extraction form was used to extract the following data from the articles included: author, year of publication, sample size, a brief presentation of the intervention that was used in each article and group, outcomes, and the differences reported between the two groups and within each group.

### 2.4. Quality Assessment

The methodological quality of the included studies was independently assessed by two authors (I.P. and A.K.), and any differences were resolved by consensus. The Physiotherapy Evidence Database (PEDro) scale, which is valid and reliable [12,13], was chosen as the tool for assessing the methodological quality of the studies in this systematic review. It contains 11 criteria, 10 of which are answered with a yes or no response. If the criterion is satisfied, then it is scored as 1 point, and if not, then it is scored as 0. Criterion 1 affects external validity and does not contribute to the final PEDro scale score. ‘Low-quality’ studies are defined as those scoring 0–3 points, while they are ‘moderate quality’ and ‘high quality’ if they score 4–6 points and 7–10 points, respectively [13].

### 2.5. Data Synthesis and Analysis

Review Manager software by the Cochrane Collaboration (RevMan Web) was used to summarize the effects of low-medium and high-intensity IMT. High-intensity IMT was considered to be when the training intensity is set to ≥50% MIP, and we chose this cut-off value to distinguish low-medium- and high-intensity IMT [14,15]. Subgroup analysis was performed if there was clinical heterogeneity in the intervention and other details of the studies, like the population characteristics for each of the two training intensities. Studies were not categorized based on the follow-up time points since all included studies analyzed the short-term effectiveness, comparing the pre- and post-intervention period between-group differences.

Quantitative synthesis was carried out in accordance with the Cochrane Handbook for Systematic Reviews of Interventions guidelines using the pre-post means and standard deviations from each chosen study for the between-group comparisons, which were either extracted directly from the articles or calculated where necessary [16]. Since the studies employed the same outcomes for the reported comparisons, the mean difference (MD) and 95% confidence intervals (CIs) were used. To determine the clinical relevance of the treatment for each outcome, a random-effects inverse variance model was chosen for meta-analysis. The I^2^ statistic was used as a measure of heterogeneity, with values greater than 50% interpreted to indicate significant heterogeneity [17].

## 3. Results

### 3.1. Identification and Description of Studies

**Figure 1:**
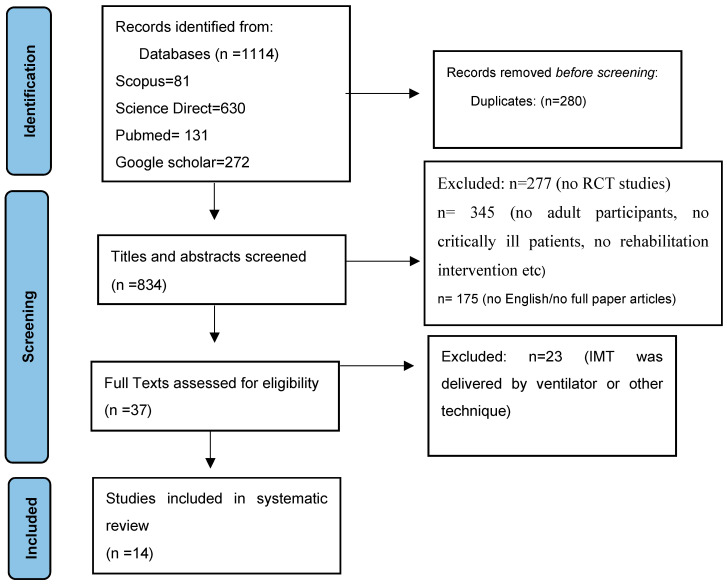
Prisma flow diagram.

From this literature search, we were able to identify 1114 studies. After excluding duplicates (*n* = 180), we screened the titles and abstracts from the remaining records. A total of 14 RCTs were finally included in this systematic review. A detailed flowchart is provided in Figure 1.

In this systematic review, 895 ICU patients were included. Seven studies [18,19,20,21,22,23,24] implemented a low-medium-intensity training program, and seven studies [25,26,27,28,29,30,31] had a high-intensity one. All studies are described in Table 1(a, b). In most studies, the intervention was initiated during the weaning period to assess the facilitation of the procedure. Only three studies [20,21,29] included tracheostomized patients to assess effectiveness in prolonged ventilation patients. Bissett et al. [28,31] mentioned the use of specialized connectors in the case of tracheostomized patients but without stating the exact number of them. Respiratory failure was the main diagnostic category of the included patients in half of the studies but without stating its etiology. Additionally, in terms of the patient’s admittance diagnostic category, sepsis was the second one, and surgical procedures was the third one.

Inspiratory muscle training was performed through threshold devices, namely analogue or electronic ones. The characteristics of the program (Table 1(b)) varied across the included study in terms regarding the duration of the program and the timeline of its initiation. Also, in two studies [21,26], we noticed the use of electronic threshold IMT devices that were designed to match the dynamic changes of the inspiratory muscle strength throughout the inspiratory effort and could automatically adapt to it.

In the control group, patients received standard physiotherapy, which in most cases included respiratory (chest) physiotherapy and mobilization. Only in the study by Hollebeke et al. [26] did we find the application of low-intensity IMT at 10% MIP.

### 3.2. Methodological Quality

The methodological quality scores of all included studies were rated with the PEDro scale (Table 2), and on average, this was found to be 5.5/10. Specifically, seven studies were rated 3–5/10, two were 6/10, four were 7/10, and one was 9/10.

**Figure 2:**
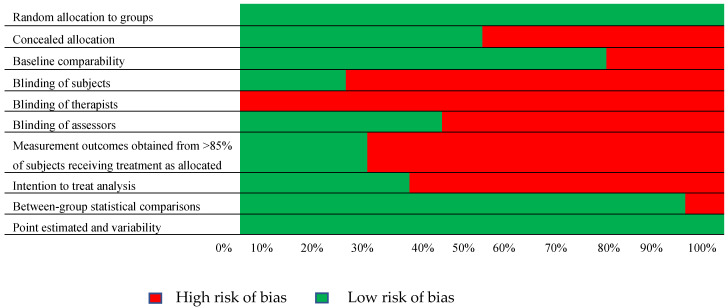
Resulting risk of bias per methodological quality item assessed with the PEDro scale.

To address the risk of bias through the methodological quality of the included studies, we examined the 10 components of the PEDro scale individually, as presented in Figure 2. There were significant sources of bias [32]. Only one category—therapist blinding—was not addressed by all of the studies. Increased risk of bias was also presented by the following categories: measurement of outcomes obtained from >85% of subjects receiving treatment as allocated (72%) and blinding of the subjects (78%).

### 3.3. Intervention Comparability

All of the included studies were randomized, included a control group, and had an adequate number of individuals. Only two studies [20,27] had a relatively low number of participants, with most ranging between 40 and 100. Sample size calculation was performed in six studies [24,25,26,28,29,30].

Although significant clinical heterogeneity was noted between the included studies, attributed to (1) variability in the intervention, (2) duration, and (3) outcomes assessed between studies, a quantitative synthesis was also performed where possible (Figure 2).

### 3.4. Effect of IMT on Maximal Inspiratory Pressure

#### 3.4.1. Effect of Low-Medium IMT on Maximal Inspiratory Pressure (Figure 3)

The effect of low-medium IMT (LM-IMT) with or without other parallel interventions on the MIP in relation to standard physiotherapy, calculated in cm H_2_O, was evaluated in 5 studies including 224 participants in total. A mean difference (MD (95% CI) = 5.36 (0.10–10.61) cm H_2_O) favoring LM-IMT with marginal, non-statistical significance (Z = 2.00, *p* = 0.05) and considerable statistical heterogeneity (I^2^ = 83, *p* = 0.0001) was noted, based on a 4.4 PEDro quality score on average (Table 2).

**Figure 3:**
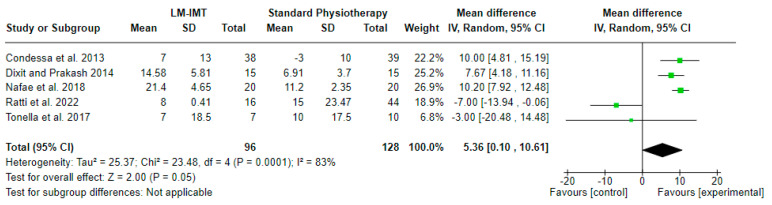
Effect of low-medium -IMT on maximal inspiratory pressure (in cm H_2_0).

#### 3.4.2. Effect of High-IMT on Maximal Inspiratory Pressure (Figure 4)

The effect of high IMT (H-IMT) with or without other parallel interventions on the MIP in relation to standard physiotherapy, calculated in cm H_2_O, was evaluated in 4 studies including 316 participants in total. A mean difference (MD (95% CI) = 7.6 (from −1.45 to 16.64) cm H_2_O) favoring H-IMT with no statistical significance (Z = 1.65, *p* = 0.10) and considerable statistical heterogeneity (I^2^ = 90, *p* < 0.00001) was noted, based on a 6 PEDro quality score on average (Table 2).

**Figure 4:**
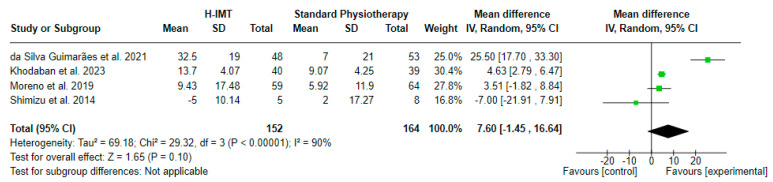
Effect of high IMT on maximal inspiratory pressure (in cm H_2_0).

### 3.5. Effect of IMT on Weaning Duration

#### 3.5.1. Effect of Low-Medium IMT on Weaning Duration (Figure 5)

The effect of low-medium IMT (LM-IMT) with or without other parallel interventions on the weaning duration in relation to standard physiotherapy, calculated in days, was evaluated in 5 studies including 224 participants in total. A mean difference (MD (95% CI) = −1.68 (from −2.97 to −0.38) days) favoring LM-IMT with statistical significance (Z = 2.54, *p* = 0.01) and substantial statistical heterogeneity (I^2^ = 60, *p* = 0.04) was noted, based on a 4.4 PEDro quality score on average (Table 2).

**Figure 5:**
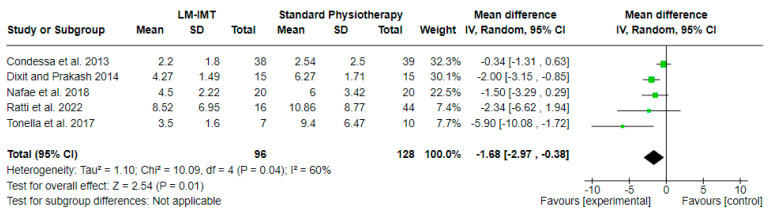
Effect of low-medium IMT on weaning duration (in days).

#### 3.5.2. Effects of High IMT on Weaning Duration (Figure 6)

The effect of high IMT (H-IMT) with or without other parallel interventions on the weaning duration in relation to standard physiotherapy, calculated in days, was evaluated in 3 studies including 215 participants in total. A mean difference (MD (95% CI) = −1.42 (from −3.72 to 0.89) days) with no statistical significance (Z = 1.20, *p* = 0.23) and considerable statistical heterogeneity (I^2^ = 99, *p* < 0.00001) was noted, based on a 6.7 PEDro quality score on average (Table 2).

**Figure 6:**
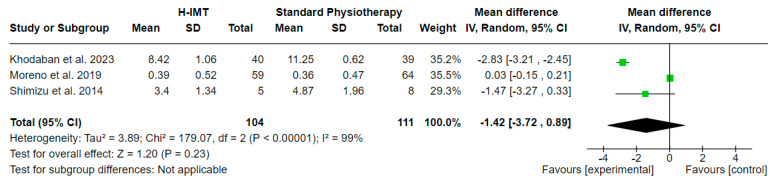
Effect of high IMT on weaning duration (days).

### 3.6. Effect of IMT on Duration of Mechanical Ventilation

#### 3.6.1. Effect of Low-Medium IMT on Duration of Mechanical Ventilation (Figure 7)

The effect of low-medium IMT (LM-IMT) with or without other parallel interventions on the weaning duration in relation to standard physiotherapy, calculated in days, was evaluated in 4 studies including 174 participants in total. A mean difference (MD (95% CI) = −3.68 (from −8.13 to 0.78) days) favoring LM-IMT with no statistical significance (Z = 1.62, *p* = 0.11) and considerable statistical heterogeneity (I^2^ = 93, *p* < 0.00001) was noted, based on a 4.8 PEDro quality score on average (Table 2).

**Figure 7:**
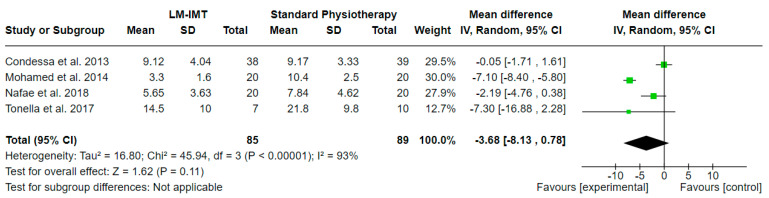
Effect of low-medium-IMT on mechanical ventilation duration (in days).

#### 3.6.2. Effect of High IMT on Weaning Duration (Figure 8)

The effect of high IMT (H-IMT) with or without other parallel interventions on the weaning duration in relation to standard physiotherapy, calculated in days, was evaluated in 4 studies including 263 participants in total. A mean difference (MD (95% CI) = 0.05 (from −2.40 to 2.50) days) with no statistical significance (Z = 0.04, *p* = 0.97) and substantial statistical heterogeneity (I^2^ = 60, *p* = 0.06) was noted, based on a 5.5 PEDro quality score on average (Table 2).

**Figure 8:**
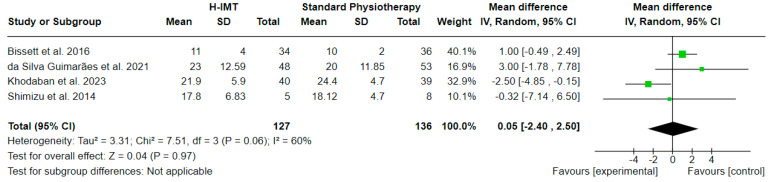
Effect of high IMT on mechanical ventilation duration (in days).

### 3.7. Effect of IMT on Rapid Swallow Breathing Index (Figure 9)

**Figure 9:**
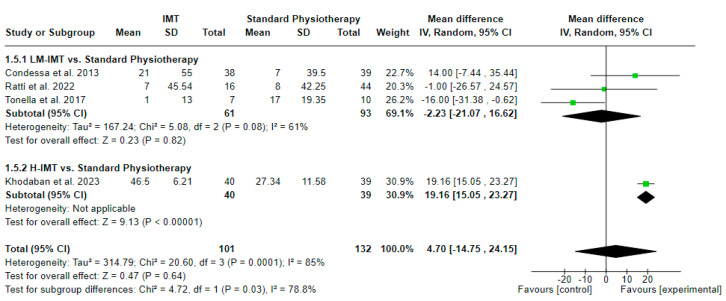
Effect of IMT on Rapid Swallow Breathing Index (RSBI) (in br/min/L).

The effect of IMT with or without parallel interventions on the Rapid Shallow Breathing Index (RSBI) in relation to standard physiotherapy was evaluated in 4 studies (3 with LM-IMT and 1 with H-IMT) including 233 participants in total. A mean difference (MD (95% CI) = 4.70 (from −14.75 to 24.15) br/min/L) favoring IMT with no statistical significance (Z = 0.47, *p* = 0.64) and considerable statistical heterogeneity (I^2^ = 85, *p* = 0.0001) was noted, based on a 5.5 PEDro quality score on average (Table 2). Yet, the effect of H-IMT tended to be greater, with the subgroup difference between LM-IMT and H-IMT (Figure 9) reaching statistical significance (*p* = 0.03).

## 4. Discussion

The aim of this systematic review and the accomplished meta-analysis was to provide novel information on the beneficial effect of low-medium- and high-intensity inspiratory muscle training in critically ill patients. Quite a few systematic reviews and meta-analyses in the past examined the advantageous use of IMT in weaning these patients, supporting the use of this intervention in clinical practice [8,9,10]. Technological innovations push the limits of rehabilitation to new boundaries, and new equipment arises to augment the therapeutic effect. From the first published study on the subject until today, there is a significant difference not only in the equipment that is used but the characteristics of the program from new data that arose from physiology studies [10,33]. Better knowledge of diaphragmatic dysfunction of ICU patients has pushed researchers and clinicians to further investigate training interventions to prevent this pathology and facilitate weaning from mechanical ventilation.

When examining the effect of either low-medium- or high-intensity programs against a standard respiratory physiotherapy program, statistically significant improvements could not be detected, although a marginal non-statistically significant difference (*p* = 0.05) was reported between LM-IMT and standard physiotherapy (Figure 3), with substantial heterogeneity noted between the compared studies (I^2^ = 83%). The considerable heterogeneity presented in the meta-analysis could explain this result. We should bear in mind the differences in the population, the time of initiation of the training, and the duration of the program between the included studies. Although we would expect that a higher intensity would lead to a strength increase, taking into consideration the principles of the strengthening exercises, that was not seen here. It likely is not just the intensity that we should consider but the volume of training that we impose on the diaphragm [34]. Also, as we withdrew the patient from excessive diaphragm unloading, and with the fear of overloading a fragile muscle, we tended to have insufficient loading. More recently, we noted that when IMT is performed with pressure threshold loading at a certain lung volume, the patient will not be able to overcome the initial loading of MIP (measured at the residual volume), especially if this is quite high. Thus, the inspiratory valve will close sooner in the breathing effort and limit the ability to perform full vital capacity inspirations [35]. This limits the loading and the training effect. Electronic devices with tapered-flow resistive loading offer a load that gradually decreases during inspiration. Thus, the applied loading remains longer, offering a greater training effect. But we do not have many studies which used this kind of training to draw a clear conclusion. This seems to be a promising technique, as pilot data from Hoffman et al. [36] demonstrated that when using tapered-flow resistive loading versus mechanical threshold loading, we can achieve a higher inspiratory volume and more breathing work with less-fatiguing muscle involvement.

Still, there is an argument regarding whether diaphragmatic dysfunction could be prevented by a strength or endurance training program [37]. Reviews that have included both strength and endurance training protocols did not point out which could be most suitable for this population [9,10]. Maybe both could be applied, but this needs to be investigated. Yet, we should not overlook the fact that the metabolic demands of exercise are not well described and understood in the ICU population, aside from efforts that have been carried out in the past few years [38]. A recent study by Jenkins et al. [39] tried to examine the metabolic demands that arise in inspiratory training under different intensities. They found significant differences in VO_2_ between the baseline and 50% negative inspiratory force (NIF) and between the baseline and 80% NIF [39]. IMT is causing a statistically significant and load-dependent increase in VO_2_ in ICU patients [39]. This shows that not all patients can exercise at high intensities. We need to be able to distinguish which patients can tolerate higher respiratory loading during IMT and which cannot.

Still, while thinking of the metabolic demands of exercise in a population that presents persistent catabolism and hypermetabolism, little attention has been given to improving muscle protein content. It is well documented that loss of muscle mass plays an important role in the development of ICU-acquired weakness [40]. Patients with a reduced diaphragm thickness will be expected to have reduced MIP and not being able to tolerate training. Nutritional strategies with high caloric feeding or even anabolic therapies are quite few in this population [40]. Yet, when we discuss matters of rehabilitation of critically ill survivors’ nutrition, this is recognized as a significant addition for recovery from muscle atrophy [41].

Regarding the duration of weaning, it is noted that LM-IMT presented a statistically significant difference in relation to the control group. In three out of five of the LM-IMT studies included in the meta-analysis, they initiated the intervention early, whilst the H-IMT ones delayed the onset. This could probably explain the difference that was noted. We should also take into consideration the variance among studies regarding the definition of weaning, which could probably have an effect, as underlined by Vorona et al. [9]. Regarding the duration of mechanical ventilation, the results remain inconclusive. Differences among the included studies regarding the weaning protocols could have contributed to this. Additionally, in the H-IMT studies that were included in the meta-analysis, all of them had different populations regarding the duration of their weaning, being prolonged or difficult. Weaning duration and success are strongly related to the level of diaphragm endurance [42]. This is not properly addressed in the included studies, and thus we do not have the data to draw certain conclusions.

This intervention has already proven its value in these outcomes and should be used in clinical practice, having considered the guidelines on the subject [8]. An older systematic review by Elkins et al. [10] reported a shorter duration of weaning but non-statistical significance.

The Rapid Shalow Breathing Index is an important and significant predictor of weaning outcomes [43]. Differences in the time of onset of IMT could affect the effectiveness of the training, as there is significant difference between prevention and rehabilitation. Differences in the durations for when patients were under controlled ventilation could alter the state of the diaphragm and its needs for recovery. In most included studies, training started before weaning onset, as this is considered to be the best approach for having a successful weaning procedure. It seems that H-IMT could potentially improve the RSBI, but the true impact on this outcome remains unclear due to limited number of studies included.

It is of high importance for clinical ICU physiotherapists to be able to recognize early patients that will have a prolonged weaning period and ICU length of stay. These patients are most likely to present ICUaw and diaphragmatic dysfunction. Although it is still debatable whether dysfunction is another feature of ICUaw, there is evidence to support that dysfunction is related to difficult weaning whilst weakness is related to prolonged ventilation [44]. Nevertheless, in both cases, there is a notable risk of an increased duration of MV. It has been also stated by Bissett et al. [45] that patients with moderate inspiratory muscle weakness (MIP ≥ 28 cm H_2_O) at the time of ventilatory independence will benefit the most from this training when we consider short-term application. Taking into consideration that even electronic devices are safe and offer a wider range of training intensity, we should consider even weaker and more fragile patients with prolonged ventilation [46]. In this meta-analysis, it was only noticed that LM-IMT has a favorable impact on the duration of weaning from mechanical ventilation. Yet, we cannot draw certain conclusions as the included studies were quite few, and the degree of heterogeneity was quite significant.

## 5. Limitations

A key limitation of this meta-analysis is the heterogeneity between studies. As this was expected, we tried to further group the studies into subgroups. Yet, this could not reflect the possibility that even low-medium-intensity studies became high with the progression of the program. There is the question of the difference in duration of the whole program and the time of initiation, especially when considering tracheostomized patients or even patients that have neurological diagnoses.

## 6. Future Directions

Inspiratory muscle training is a promising form of intervention to assist patients with weaning difficulties. In clinical practice, we need a well-structured protocol of early assessment in order to identify patients at risk, minimize control ventilation, and awaken trials that would allow the early onset of intervention even at lower intensities. Although full cooperation is needed to be able to measure the MIP, we should consider the use of diaphragmatic ultrasound as a means of early detection of diaphragmatic atrophy and weakness [47,48,49]. We also need to investigate different weaning procedures and strategies in spontaneous breathing trials, along with the use of IMT in difficult-to-wean populations. The heterogeneity that a critically ill population presents may require different approaches, and thus we do not just need to evaluate the effectiveness of IMT itself but how we can increase its effectiveness by combining it with noninvasive ventilation [50] or even high-flow nasal cannula [51].

Taking into consideration the heterogeneity of the included studies, we need large multicenter trials to be able to compare the effectiveness between different protocols and different ICU populations in relation to their weaning status.

## 7. Conclusions

IMT in ICU patients that have received mechanical ventilation is beneficial, as previous studies have noticed significant improvements in inspiratory muscle strength, duration of weaning, and duration of MV. In our studies, while examining the benefits of implementing LM-IMT or H-IMT in the above-mentioned outcomes, we did not find any significant effect aside from that of the LM-IMT in terms of weaning duration. There is a need to further investigate the differences in the applied protocols to augment their effectiveness. The training stimulus needs to be tailored to the needs of its patient, especially when considering the case of difficult or prolonged weaning. A closer monitoring of the rehabilitation trajectories of the diaphragm would also help us to better understand what this muscle needs. We need to further incorporate into our clinical practice the use of the ventilators’ waveform or even ultrasonography.

## Figures and Tables

**Table 1:** (a) Descriptions and characteristics of the included studies in the systematic review (MV = mechanical ventilation; MIP = maximal inspiratory pressure; NIP = negative inspiratory pressure; RSBI = Rapid Shallow Breathing Index; IMT = inspiratory muscle training). (b) Descriptions of the experimental groups (MIP = maximal inspiratory pressure).

**(a)**					
**RCT**	**Population**	**Intervention**	**Comparison**	**Outcome**	**Results**
**Low-Medium Intensity of IMT (<50%)**					
Condessa et al., 2013 [24]	*N* = 92 (respiratory failure)	40% MIP	Standard physiotherapy	MIP RSB Duration MV Weaning duration	*p* < 0.05 only in MIP
Ibrahiem et al., 2014 [18]	*N* = 30 (respiratory failure)	30% NΙP	Standard physiotherapy	NIP	*p* < 0.005
Mohamed et al., 2014 [19]	*N* = 40 (respiratory failure)	30% NIP	standard physiotherapy	NIP Duration MV	NIP: *p* < 0.001 MV duration: *p* < 0.001
Dixit and Prakash, 2014 [22]	*N* = 30 (general ICU)	Τhreshold: 30% MIP	Standard physiotherapy	MIP Weaning duration	MIP: *p* = 0.0009 Weaning duration: *p* = 0.0009
Tonella et al., 2017 [20]	*N* = 19 (medical)	KH2: 30% MIP	Standard physiotherapy	MIP RSBI Duration MV Weaning duration	MIP: *p* = 0.017 Weaning duration: *p* = 0.0192
Nafae et al., 2018 [23]	*N* = 40 (medical)	Threshold IMT, 9 cm H_2_O pressure	Standard physiotherapy	MIP RSBI Weaning success Duration MV Weaning duration	*p* < 0.05 in all aside Weaning success
Ratti et al., 2022 [21]	*N* = 132 (medical, surgical, trauma, neurological)	KH2: 30% MIP	Standard physiotherapy	MIP RSBI Weaning duration	*p*: ns between groups
**High Intensity of IMT (≥50%)**					
Shimizu et al., 2014 [27]	*N* = 13 (medical, surgical, trauma, neurological)	Τhreshold: 50% ΜIP	Standard physiotherapy	MIP Weaning duration Duration MV	*p* = ns between groups
Bissett et al., 2016 [28]	*Ν* = 70 (medical, surgical, neurological)	Threshold: 50% MIP	standard physiotherapy	FRI ΜΙP Dyspnea	*p* < 0.05 only in MIP
Moreno et al., 2019 [25]	*N* = 126 (medical, surgical)	Threshold: 50% MIP	Standard physiotherapy	MIP Duration MV Weaning duration Weaning success	*p* = ns between groups
da Silva Guimarães et al., 2021 [29]	*N* = 43 (medical)	Threshold IMT: 80% MIP	Standard physiotherapy	MIP	*p* < 0.001
Van Hollebeke et al., 2022 [26]	*N* = 41 (surgical, medical)	KH2: 50% MIP	10% MIP 6 sets of 6–8 breaths	MIP	*p* = ns between groups
Bissett et al., 2023 [31]	*N* = 70 (surgical, medical, neurological)	Threshold IMT: 50% MIP	Standard physiotherapy	MIP FRI Duration MV	*p* = ns between groups
Khodabandeloo et al., 2023 [30]	*N* = 79 (medical)	Threshold IMT: 50% MIP	Standard physiotherapy	MIP RSBI Weaning duration Duration MV	MIP: *p* < 0.001 RSBI: *p* < 0.001 Duration MV: *p* < 0.05 Weaning duration: *p* < 0.001
**(b)**					
**RCT**		**Intervention**		**Comparison (Standard Physiotherapy)**	
Condessa et al., 2013 [24]		Intensity: 40% MIP, 5 sets of 10 breaths		Passive to active-assisted mobilization of the limbs, chest compression, positioning	
		Frequency: 2 times/day, 7 days/week			
Ibrahiem et al., 2014 [18]		Intensity: 30% NΙP. 18 breaths, 5–6 sets		manual hyperinflation, percussion, vibrations, and muscle training (for upper and lower limbs)	
		Frequency: 2 times/d			
		Time: 10 min			
		Progression: Increase 1–2 cm H_2_0			
Mohamed et al., 2014 [19]		Intensity: 30% NIP; 18 breaths, 5–6 sets		Manual hyperinflation, percussion, vibrations, and muscle training (for upper and lower limbs)	
		Frequency: 2 times/d			
		Time: 10 min			
		Progression: Increase 1–2 cm H_2_0			
Dixit and Prakash, 2014 [22]		Intensity: threshold 30% MIP; 6 breaths, 5 sets		Expansion techniques, percussion, vibration, postural drainage, active and passive mobilization of the limbs	
		Frequency: 2 times/day, 7 days/week			
		Time: 5–30 min			
		Progression: Increase 10% ΜΙP			
Tonella et al., 2017 [20]		Intensity: threshold 30% MIP; 10 breaths, 3 sets		Nebulization sessions	
		Frequency: 2 times/day			
		Progression: increase 10% daily			
Nafae et al., 2018 [23]		Intensity: threshold IMT, 9 cm H_2_O pressure, 4 sets of 6–8 breaths		Expansion techniques, percussion, vibration, postural drainage, active and passive mobilization of the limbs	
		Duration: 30 min			
		Progression: increase 4 cm H_2_0 every session			
Ratti et al., 2022 [21]		Intensity: threshold KH2 30%MIP, 3 sets, 10 breaths		Active-assistive mobilization of the limbs, bronchial hygiene	
		Progression: daily increase 10% MIP			
Shimizu et al., 2014 [27]		Intensity: threshold 50% ΜIP; 10 breaths, 3 sets		Nebulization sessions	
		Frequency: 2 times/day, 7 days/week			
Bissett et al., 2016 [28]		Intensity: threshold 50% MIP, 5 sets, 6 breaths		Secretion clearance techniques, limb exercises, assisted mobilization	
		Frequency: 1 per day, 5 days/week, 2 weeks			
		Duration: 5–30 min			
		Progression: increase: 1–2 cm H_2_O			
Moreno et al., 2019 [25]		Intensity: threshold 50% MIP, 3 sets, 10 breaths		Chest physiotherapy, limb exercises, mobilization	
		Frequency: 2 times/day, 7 days/week			
da Silva Guimarães et al., 2021 [29]		Intensity: threshold IMT 80% MIP; 2 sets, 30 breaths		Early mobilization	
		Progression: the load increased within each set of breaths until reaching 80% MIP			
Van Hollebeke et al., 2022 [26]		Intensity: tapered-threshold 50% MIP; 6 sets of 6–8 breaths		Tapered-threshold 10% MIP 6 sets of 6–8 breaths	
		Progression: to the highest level tolerated			
Bissett et al., 2023 [31]		Intensity: threshold IMT 50% MIP; 5 sets of 6 breaths		Secretion clearance techniques	
		Frequency: once per day, 5 days/week			
		Progression: highest level tolerated to complete sixth breath			
Khodabandeloo et al., 2023 [30]		Intensity: threshold IMT 50% MIP; 5 sets of 6 breaths Frequency: 5 days/w		Passive to active movements of the limbs, chest physiotherapy (vibration and percussion), and repositioning	
		Progression: Daily increase of 10% MIP			

**Table 2:** Ratings of included studies according to PEDro scale. (* item not included in total score).

Study	1	2	3	4	5	6	7	8	9	10	11	Score
Ibrahiem et al., 2014 [18]	✓ *	✓	−	✓	−	−	−	✓	✓	✓	✓	6/10
Mohamed et al., 2014 [19]	✓ *	✓	_	✓	_	_	_	_	_	✓	✓	4/10
Dixit and Prakash, 2014 [22]	✓ *	✓	−	−	−	−	−	−	−	✓	✓	3/10
Bissett et al., 2016 [28]	✓ *	✓	✓	✓	−	−	✓	_	✓	✓	✓	7/10
Tonella et al., 2017 [20]	✓ *	✓	✓	✓	−	−	−	✓	−	✓	✓	6/10
Nafae et al., 2018 [23]	✓ *	✓	_	✓	_	_	_	_	_	✓	✓	4/10
Ratti et al., 2022 [21]	✓ *	✓	✓	_	_	_	_	_	_	✓	✓	4/10
Condessa et al., 2013 [24]	✓ *	✓	✓	✓	_	_	✓	_	_	_	✓	5/10
Shimizu et al., 2014 [27]	✓ *	✓	−	✓	−	−	−	−	−	✓	✓	4/10
Moreno et al., 2019 [25]	✓ *	✓	✓	✓	✓	−	✓	✓	✓	✓	✓	9/10
da Silva Guimarães et al., 2021 [29]	✓ *	✓	_	_	_	_	_	✓	_	✓	✓	4/10
Van Hollebeke et al., 2022 [26]	✓ *	✓	✓	✓	✓	-	✓	-	-	✓	✓	7/10
Bissett et al., 2023 [31]	✓ *	✓	✓	✓	_	_	✓	_	✓	✓	✓	7/10
Khodabandeloo et al., 2023 [30]	✓ *	✓	-	✓	✓	-	✓	-	✓	✓	✓	7/10

## Data Availability

Data are unavailable due to privacy or ethical restrictions.
